# A Longitudinal Survey of Postgraduate Residency Hospital Type and Career Paths in Japan (1996–2016)

**DOI:** 10.7759/cureus.15711

**Published:** 2021-06-17

**Authors:** Masatoshi Ishikawa

**Affiliations:** 1 International Health, Harvard T.H. Chan School of Public Health, Boston, USA

**Keywords:** public health, medical education and training, human resource management, national physician census, longitudinal study

## Abstract

Objective

To analyze the relationship between postgraduate residency hospital type and career paths in Japan.

Methodology

A longitudinal study based on secondary data collected from the national physician census was conducted in Japan. The sample comprised 3,991 residents for the 1996-2006 cohort and 6,153 residents for the 2004-2014 cohort.

Results

The percentage of residents who trained at university hospitals in their first year of registration decreased dramatically from 70% to 40% due to the 2004 mandatory clinical training reform. In contrast, the percentage of physicians working at university hospitals in their third year of registration increased from 20% to around 40%. Further, the number of physicians who had not worked at university hospitals during their first 10 years increased from 12% in 2004 to 18% in 2014.

Conclusions

Since the introduction of the mandatory postgraduate clinical training, residents’ career paths have changed based on the residency hospital type that they attended. The resident shortage at university hospitals remains a longstanding issue in Japan.

## Introduction

Continuous training of high-quality physicians through ongoing medical education is essential to maintaining a healthcare system capable of providing high-quality medical care. Medical education consists of three successive phases: undergraduate medical education, postgraduate medical education, and lifelong professional development [1]. Policy experts must continually validate and consider revising this framework considering the state of medical education and the resulting achievements of the participating doctors.

Postgraduate medical education begins after the medical school curriculum is completed and a national certification examination is passed. This period allows residents to develop a wide range of diagnostic and therapeutic skills under the supervision of mentors, and it also serves as a preparatory period for residents to acquire a specialist license [1]. To ensure consistency in the quality of postgraduate medical education programs, many countries have enacted national regulations, and the World Federation for Medical Education has published global standards for postgraduate medical education [1].

In 2004, Japan’s Ministry of Health, Labour and Welfare (MHLW) made postgraduate clinical training mandatory for doctors who graduate medical school and pass the national licensing examination with the intent of providing residents with basic diagnostic and therapeutic skills to properly treat the common injuries and diseases encountered in routine practice, regardless of their intended future specialization [2].

The newly mandated course is a two-year postgraduate clinical training program. Initially, the standard curriculum involved rotations between internal medicine, surgery, emergency medicine (including anesthesiology), obstetrics/gynecology, pediatrics, psychiatry, and community health departments [2]. In 2010, surgery, anesthesiology, obstetrics/gynecology, pediatrics, and psychiatry were changed to compulsory electives. Starting in 2020, there are plans to re-institute mandatory rotations in all except anesthesiology [3].

Japanese doctors followed similar career paths before this training system became compulsory [4]. Once licensed, doctors joined different departments in a medical university and received specialist training. Even after specializing, doctors would continue to improve their skills by gaining experience in basic research and roles at affiliated hospitals (i.e., institutions related to a medical university, which can request physicians of specific departments to be assigned to meet local needs). Such university networks in Japan, called “medical offices” (ikyoku), exist to minimize the uneven distribution of physicians by assigning doctors to communities in need.

Since 2004, doctors have increasingly elected to pursue postgraduate clinical training at non-university hospitals [5]. One study claims that this trend worsened the already poor regional distribution of physicians, which compromised the ability of university medical offices to guarantee an appropriate number of doctors on site, especially in rural areas, which disrupted the established dispatch networks [6]. Another study concluded that the change exacerbated the migration of doctors to areas that already had high physician density, especially urban locations [7].

Several reports claim that the 2004 reform has worsened the already uneven regional distribution of doctors [7-10]. However, the impacts of the increasing residency rates at non-university hospitals on doctors’ subsequent career paths, and specifically on the type of institution they decide to work at after concluding their training, have not been examined.

Prior to 2004, doctors trained at university and non-university hospitals and pursued careers in different types of medical institutions (university hospitals, clinics, etc.) at consistent rates after completing their residency [11]. However, no study has documented the distribution of hospital type after the reform was enacted. Data from an MHLW survey of doctors following completion of their postgraduate clinical training indicate that about 50% wished to work at a university hospital after residency, with more female than male doctors preferring university hospital employment [12]. It is unclear, however, how the introduction of mandatory clinical training has changed the patterns of doctors’ post-residency employment.

This study aimed to investigate how mandatory postgraduate clinical training has affected the subsequent career paths of young physicians by comparing the attributes of cohorts trained before and after its introduction. Special attention was paid to whether and how residents’ subsequent career paths diverge based on institution type; this was examined by comparing cohorts trained at university versus non-university hospitals. The results are expected to inform policy recommendations for medical education in the future.

This article was previously published as a preprint on Research Square on June 29, 2020.

## Materials and methods

This study was approved by the institutional review board of the Harvard T.H. Chan School of Public Health (Approval No. 18-1422). The requirement for informed consent was waived as the national survey was mandatory according to medical law.

The MHLW conducts a national census survey of physicians, dentists, and pharmacists every two years. Japan’s Medical Practitioners’ Act requires physicians to report their status, and the response rate is approximately 90% [13]. Using this data, a retrospective longitudinal study based on secondary data analysis gathered from 1996 to 2016 was conducted. The survey data included physicians’ identification (registration number), year enrolled in the national registry, age, sex, and workplace type (municipality and institution).

In 2016, 344 secondary medical areas were classified into three groups based on a combination of population size and density: urban, intermediate, and rural. Institutions were classified as university hospital, non-university hospital, clinic, and others.

First, the proportions of first- and third-year doctors working at each type of institution between 1996 and 2016 during the surveyed years were tabulated and presented. Next, employment data for two cohorts were analyzed: (1) doctors newly licensed in 1996, the earliest dataset available, and (2) doctors newly licensed in 2004, the year that postgraduate clinical training became compulsory. Specifically, trends in institutional employment through 2016 were tabulated and presented for both groups.

Members of these two cohorts (1996, 2004) were divided into two groups based on whether their first year as a licensed doctor was spent at a university or a non-university hospital. The cohort attributes were compared at study enrollment year and 10 years later (i.e., 1996 vs. 2006, 2004 vs. 2014) and tabulated by sex, age, region, “current prefecture” (i.e., whether they continued to practice in the same prefecture after 10 years), and institution type. Similar comparisons were made for the cohort subgroups of doctors who had never worked at a university hospital during the survey period.

For the statistical analysis with Stata version 15.1 (StataCorp, College Station, TX), P-values (two-tailed) less than 0.05 were considered significant.

## Results

Distribution of physicians in their first year and third year as licensed physicians

**Figure 1:**
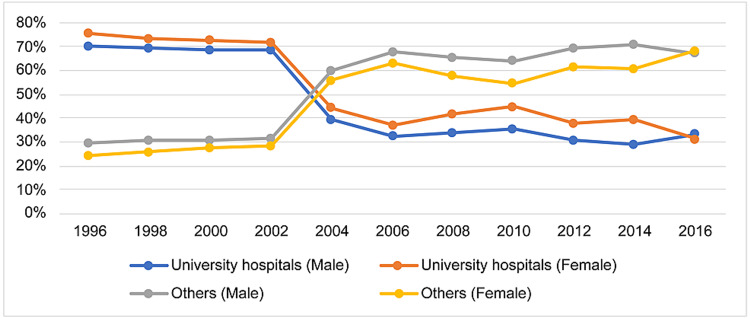
Institutional trends in physicians’ first year in the national registry.

Figure 1 depicts the changes in the percentages of doctors training at each institution type in their first year as licensed physicians. From 1996 to 2002, around 70% of residents were training at university hospitals; this rate declined sharply in 2004 and has hovered around 30-40% ever since. At all survey time points, a greater proportion of female doctors were training at university hospitals compared to their male peers.

**Figure 2:**
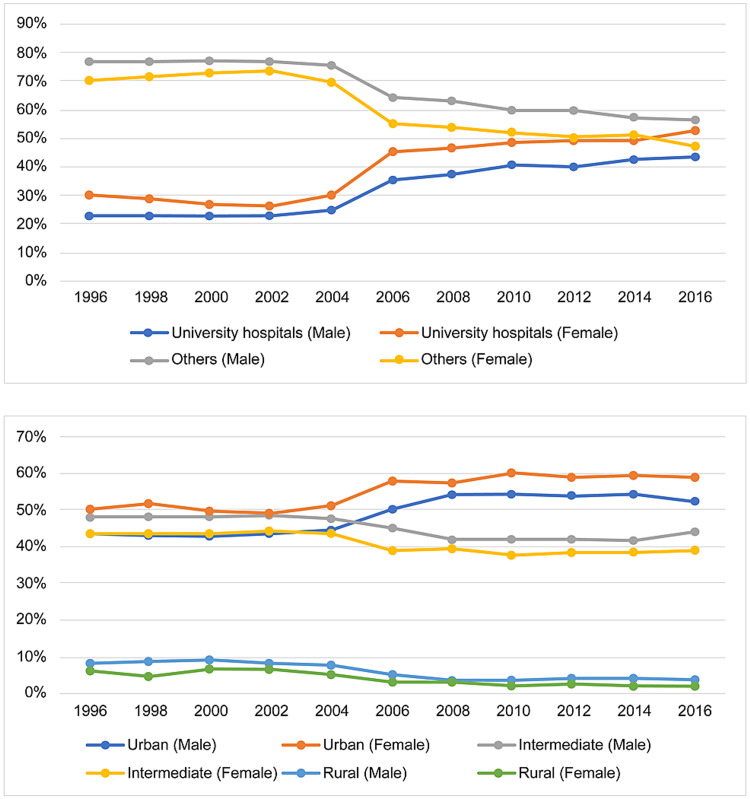
Institutional trends in physicians’ third year in the national registry.

Figure 2 depicts changes in the percentages of doctors training at each institution type in their third year as licensed physicians. From 1996 to 2004, the percentage training at university hospitals hovered around 20-30%; in 2006, it increased to around 40% and has been on an upward trend ever since.

Institutional trends among physicians licensed in 1996 and 2004 (until 2016)

**Figure 3:**
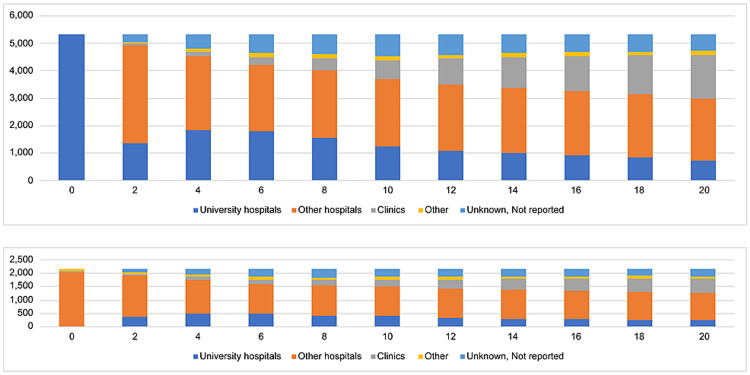
Institutional trends of physicians licensed in 1996 (1996-2016).

Figure 3 depicts the changes in the distribution of institutions employing newly licensed physicians in 1996 through 2016. In 2006, 10 years after registration, 23% of doctors who had trained in university hospitals in their first year as licensed physicians were still working at university hospitals; 47% had moved to other hospitals, and 13% to clinics. In 2016, 20 years after their registration, 14% of the cohort was still working at university hospitals compared with 42% at other hospitals and 30% at clinics. In 2006, 10 years after their registration, 19% of doctors who had trained in non-university hospitals in their first year as licensed physicians had moved to work at university hospitals; 52% were working at other hospitals, and 17% at clinics. In 2016, 20 years after their registration, 11% of the cohort was working at university hospitals compared with 48% at other hospitals and 24% at clinics.

**Figure 4:**
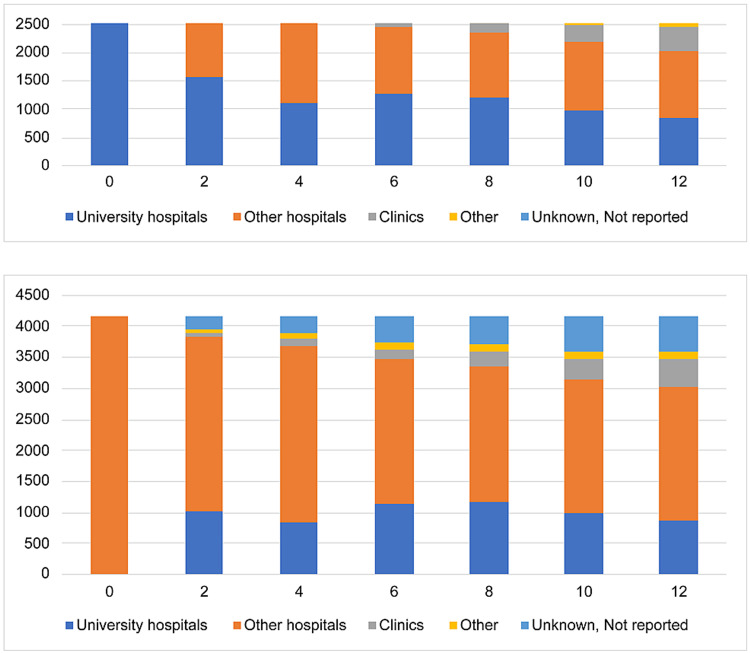
Institutional trends of physicians licensed in 2004 (2004-2016).

Figure 4 depicts the changes in the distribution of institutions employing newly licensed physicians in 2004 through 2016. In 2014, 10 years after their registration, 34% of doctors who had trained in university hospitals in their first year as licensed physicians were still working at university hospitals, 41% had moved to other hospitals, and 10% to clinics. In 2016, 12 years after their registration, 29% of the cohort was working at university hospitals compared with 41% at other hospitals and 15% at clinics. In comparison, in 2014, 10 years after their registration, 24% of doctors who had trained in non-university hospitals in their first year as licensed physicians had moved to work at university hospitals; 24% were working at other hospitals, and 52% at clinics. In 2016, 12 years after their registration, 21% of the cohort was working at university hospitals compared with 52% at other hospitals and 10% at clinics.

Physicians’ 10-year career choices and comparison of two registration cohorts

Table 1 details the attributes of physicians examined in 1996 and 2006 and groups them into those who did their first year of residency at university versus non-university hospitals. Overall, 12% of physicians licensed in 1996 and 42% of those trained at non-university hospitals in their first year in the registry had never worked at a university hospital during the survey period. By institution type, a greater percentage of the cohort initially trained at university hospitals was working in clinics in 2006. By age group, doctors aged 40 years and older accounted for the highest percentage of the cohort with no university hospital experience during the survey period. By region, doctors working in rural areas accounted for the highest percentage of the cohort with no university hospital experience during the survey period. More doctors who trained at university hospitals in their first year on the registry remained in the same prefecture than had moved away.

**Table 1:** Distribution of physicians 10 years after registration (1996 cohort, 2006 survey).

	Trained in postgraduate education hospitals					
	Trained in university hospitals		Total	No experience in university hospital		
Proportion trained at postgraduate education hospital	2,825	70.8%	1,166	29.2%	484	12.1%
Sex, n, %						
Male	2,333	82.6%	999	85.7%	409	84.5%
Female	492	17.4%	167	14.3%	75	15.5%
Age, n, %						
≦39	0	0.0%	0	0.0%	0	0.0%
40-54	2,739	97.0%	1,110	95.2%	438	90.5%
55-69	85	3.0%	53	4.5%	43	8.9%
≧70	1	0.0%	3	0.3%	3	0.6%
Workplace, n, %						
Urban	1,235	43.7%	488	41.9%	193	39.9%
Intermediate	1,393	49.3%	588	50.4%	241	49.8%
Rural	197	7.0%	90	7.7%	50	10.3%
Current prefecture						
Same as year of registration	1,739	61.6%	658	56.4%	278	57.4%
Different	1,086	38.4%	508	43.6%	206	42.6%
Type of institution, n, %						
Clinic	859	30.4%	297	25.5%	159	32.9%
Academic hospital	377	13.3%	130	11.1%	0	0.0%
Other hospital	1,535	54.3%	704	60.4%	301	62.2%
Others	54	1.9%	35	3.0%	24	5.0%

Table 2 details the attributes of physicians examined in 2004 and 2014, grouping them into categories for those who did their first year of residency at university versus non-university hospitals. Overall, 18% of physicians licensed in 2004 and 42% of those trained at non-university hospitals in their first year in the registry had never worked at a university hospital during the survey period. By institution type, a greater percentage of the cohort initially trained at university hospitals was working in clinics in 2006. By age group, doctors aged 40 years and older accounted for the highest percentage of the cohort with no university hospital experience during the survey period. By region, doctors working in rural areas accounted for the highest percentage of the cohort with no university hospital experience during the survey period. More doctors trained at university hospitals in their first year on the registry remained in the same prefecture than moved away.

**Table 2:** Distribution of physicians 10 years after registration (2004 cohort, 2014 survey).

	Trained in postgraduate education hospitals					
	Trained in university hospitals		Total	No experience in university hospital		
Proportion trained at postgraduate education hospital	2,543	41.3%	3,610	58.7%	1,134	31.4%
Sex, n, %						
Male	1,714	67.4%	2,560	70.9%	819	72.2%
Female	829	32.6%	1,050	29.1%	315	27.8%
Age, n, %						
≦39	2,267	89.1%	3,187	88.3%	942	83.1%
40-54	270	10.6%	405	11.2%	181	16.0%
55-69	6	0.2%	18	0.5%	11	1.0%
≧70	0	0.0%	0	0.0%	0	0.0%
Workplace, n, %						
Urban	1,357	53.4%	1,901	52.7%	569	50.2%
Intermediate	1,073	42.2%	1,542	42.7%	495	43.7%
Rural	113	4.4%	167	4.6%	70	6.2%
Current prefecture						
Same as year of registration	1,632	64.2%	1,911	52.9%	606	53.4%
Different	911	35.8%	1,699	47.1%	528	46.6%
Type of institution, n, %						
Clinic	287	11.3%	331	9.2%	143	12.6%
Academic hospital	994	39.1%	975	27.0%	0	0.0%
Other hospital	1,200	47.2%	2,167	60.0%	942	83.1%
Others	62	2.4%	137	3.8%	49	4.3%

## Discussion

The percentage of residents who had trained at university hospitals in their first year of registration dramatically decreased from 70% to approximately 40% due to the 2004 reform. Contrarily, after the mandatory clinical training, the percentage of physicians who had worked at university hospitals in their third year of registration increased from 20% to about 40% in 2004. The percentage of physicians who had never worked at a university hospital in their first 10 years on the registry increased after the reform from 12% in 2004 (1994 cohort) to 18% in 2014 (2004 cohort).

The shortage of residents at university hospitals is a longstanding challenge in healthcare. Policy experts have also noted that after the 2004 reform, the percentage of residents who trained at university hospitals in their first year of registration dropped from 70% to 40% [6]. The study findings demonstrate that this trend has continued. In contrast, the percentage of physicians working at university hospitals in their third year of registration increased to around 40% in the year of the reform and has generally shown an upward trend since then. This may be because some doctors decided to relocate to university hospitals after postgraduate clinical training to join medical offices at the same time that they started a specialist residency program [4]. In addition, shortages of young doctors at university hospitals compromise the ability of these hospitals to dispatch staff to affiliated hospitals [6], reflecting the heightened tendency of doctors belonging to medical offices to be placed in university hospitals.

Analysis of data from the period prior to the introduction of the mandatory postgraduate clinical training show that doctors chose to work at different institution types at consistent rates, regardless of whether they spent their first year as licensed physicians at a university or non-university hospital [11]. This study, in contrast, demonstrates that residents’ subsequent career paths diverged depending on where they trained in their first year of registration. For example, the percentage of doctors who had never worked at a university hospital in their first 10 years on the registry increased after the reform from 12% in 2004 (1994 cohort) to 18% in 2014 (2004 cohort).

This study is the first to reveal that following the introduction of the mandatory postgraduate clinical training, there was an increase in the percentage of Japanese physicians with no university hospital experience. As suggested in previous studies, this trend could partially be a result of the decrease in new members of medical offices in universities [12]. Observers have indicated that residency at a non-university hospital is superior to a university hospital residency in several respects, including the residency system, clinical skills acquired, and salary [13]. However, one of the advantages of a university hospital residency is the ease of conducting research. Prior studies have demonstrated that residents’ participation in research activities is related to higher levels of satisfaction with residency training [14]. Consequently, increasing number of physicians who are not associated with universities may weaken the medical research ability of Japanese physicians.

The strength of the present study is that it has used individual data from the national census; therefore, the sample size was large and the capture rate was high. However, there are several limitations. First, the characteristics of physicians were self-reported, therefore, misclassification may have occurred. This may also lead to recall bias and measurement or reporting bias due to unreliable self-reporting. Second, the results cannot be generalized as the study suffers from the limitations and risk of bias inherent in retrospective studies and questionnaire and registry studies. Third, we did not obtain data to investigate part-time physicians. Fourth, only quantitative analysis was conducted. The use of interviews and questionnaires could facilitate more comprehensive research.

## Conclusions

Since the introduction of the mandatory postgraduate clinical training in 2004, residents’ career paths have changed dramatically based on the residency hospital type they attended. The shortage of residents at university hospitals remains a longstanding issue in Japan. However, increasing the number of physicians who are not associated with universities may weaken the medical research ability of Japanese physicians.
